# Contact Mechanics of Elliptical and Spherical Head Implants during Axial Rotation in Anatomic Total Shoulder Arthroplasty: A Biomechanical Comparison

**DOI:** 10.3390/jcm12154918

**Published:** 2023-07-26

**Authors:** Lukas N. Muench, Maria Slater, Simon Archambault, Daniel P. Berthold, Marco-Christopher Rupp, Elifho Obopilwe, Mark P. Cote, Augustus D. Mazzocca

**Affiliations:** 1 Department of Sports Orthopaedics, Technical University of Munich, 81675 Munich, Germany; 2 Department of Orthopaedic Surgery, UConn Health, Farmington, CT 06030, USAmcote@uchc.edu (M.P.C.); 3 Department of Orthopaedics and Trauma Surgery, Musculoskeletal University Center Munich (MUM), University Hospital, Ludwig Maximilian University Munich, 82152 Munich, Germany; 4 Massachusetts General Hospital, Massachusetts General Brigham, Harvard Medical School, Boston, MA 02115, USA

**Keywords:** humeral head, elliptical, spherical, implant design, total shoulder arthroplasty, contact mechanics

## Abstract

Background: Elliptical humeral head implants have been proposed to result in more anatomic kinematics following total shoulder arthroplasty (aTSA). The purpose of this study was to compare glenohumeral contact mechanics during axial rotation using spherical and elliptical humeral head implants in the setting of aTSA. Methods: Seven fresh-frozen cadaveric shoulders were utilized for biomechanical testing in neutral (NR), internal (IR), and external (ER) rotation at various levels of abduction (0°, 15°, 30°, 45°, 60°) with lines of pull along each of the rotator cuff muscles. Each specimen underwent the following three conditions: (1) native, and TSA using (2) an elliptical and (3) spherical humeral head implant. Glenohumeral contact mechanics, including contact pressure (CP; kPa), peak contact pressure (PCP; kPa), and contact area (CA; mm^2^), were measured in neutral rotation as well as external and internal rotation using a pressure mapping sensor. Results: Elliptical head implants showed a significantly lower PCP in ER compared to spherical implants at 0° (Δ−712.0 kPa; *p* = 0.034), 15° (Δ−894.9 kPa; *p* = 0.004), 30° (Δ−897.7 kPa; *p* = 0.004), and 45° (Δ−796.9 kPa; *p* = 0.010) of abduction, while no significant difference was observed in ER at 60° of abduction or at all angles in NR and IR. Both implant designs had similar CA in NR, ER, and IR at all tested angles of abduction (*p* > 0.05, respectively). Conclusions: In the setting of aTSA, elliptical heads showed significantly lower PCP during ER at 0° to 45° of abduction, when compared to spherical head implants. However, in NR and IR, PCP was similar between implant designs. Both designs showed similar CA during NR, ER, and IR at all abduction angles. Level of Evidence: basic science; controlled laboratory study.

## 1. Introduction

Anatomic total shoulder arthroplasty (aTSA) has been proven to be a reliable treatment option for patients with end-stage glenohumeral osteoarthritis (OA), following the major guiding principle of restoring the native anatomic relationship of the glenohumeral joint [1,2,3]. Traditionally, the native glenohumeral joint has been considered to be an articulation of two perfectly spherical components, which has significantly influenced implant design in the past [4,5,6,7].

However, more recent anatomic studies of the shoulder have challenged this paradigm and shown that the humeral head is rather elliptical than spherical in shape [8,9,10,11,12]. As implants more closely restoring the native anatomy may ensure more physiological joint kinematics and superior durability, these findings have questioned if using a spherical arthroplasty design is most suitable to replicate the native humeral head [13]. Accordingly, biomechanical investigations have shown that elliptical head implants more accurately restored native shoulder kinematics in terms of glenohumeral translation and rotational range of motion [13,14,15]. Further, a joint replacement using elliptical heads was found to provide greater total resection and cortical coverage than implants with a spherical shape, while avoiding excessive overhang [16].

More importantly, these biomechanical observations have also been reflected in recent clinical studies [17,18]. A radiographic evaluation of patients undergoing aTSA found that an elliptical humeral head implant design most closely reproduced the geometry of the native humeral head when compared to a spherical design [18]. In addition, stemless aTSA using a novel multiplanar osteotomy and elliptical humeral head implant achieved greater early range of motion compared with standard aTSA [17].

Although these previous evaluations indicate favorable properties of elliptical-shaped humeral head implants in the setting of aTSA, evidence pertaining to the effect of humeral head implant shape on glenohumeral contact mechanics during shoulder motion remains scarce. As the elliptical head design is characterized by a flatter surface, this may be expected to result in a better distribution of contact pressure between the articulating components, which is a factor critical to implant durability and ultimately arthroplasty survivorship [19].

Thus, the purpose of the study was to compare glenohumeral contact mechanics during axial rotation using spherical and elliptical humeral head implants in the setting of aTSA. It was hypothesized that the spherical head design would result in a significantly higher contact pressure when compared to the elliptical design.

## 2. Materials and Methods

Seven fresh-frozen cadaveric shoulders with a mean age of 66.3 ± 7.8 years were used for the study (Science Care Inc., Phoenix, AZ, USA). All specimens underwent visual and radiographic inspection to exclude those with tears of the rotator cuff tendons and capsule, moderate to severe osteoarthritis, bony defects, or joint contractures. As de-identified specimens were not considered to constitute human subjects research, prior Institutional Review Board approval was not required.

### 2.1. Specimen Preparation

After having been thawed overnight at room temperature, specimens were dissected free of skin, subcutaneous tissue, and muscles. Rotator cuff muscles, capsule, and the coracoacromial ligament were carefully preserved. Under fluoroscopy control (Mini C-Arm, GE Medical Systems Inc., Waukesha, WI, USA), a 2.0 mm K-wire was drilled parallel to the glenoid surface from posterior to anterior at the middle of the superior–inferior diameter. A second 2.0 mm K-wire was drilled from inferior to superior parallel to the glenoid. The scapulae were trimmed using an oscillating saw and potted in a custom rectangular box with the glenoid surface being aligned parallel to the floor. After being shortened, the humerus was centered and potted in a poly-vinyl chloride pipe (PVC; diameter, 3.8 cm; length, 7 cm) using bone cement, leaving only 2 cm of the proximal humeral shaft exposed, in order to minimize diaphyseal bending moments [20,21,22].

### 2.2. Testing Setup

**Figure 1:**
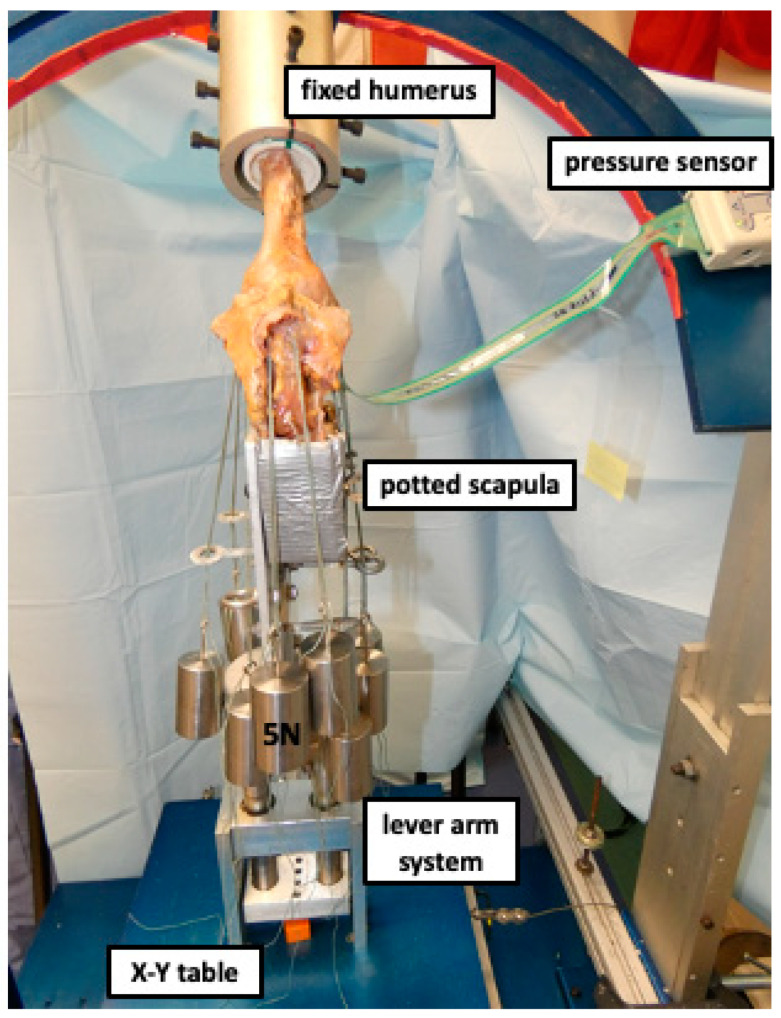
Displaying a right shoulder specimen mounted to the shoulder testing rig in 60° of abduction. The scapula is fixed to a vertical linear bearing translator and lever arm system on top of an X-Y table, allowing for glenohumeral translation in the anteroposterior and superoinferior directions. During testing, an axial compression load of 40 N was constantly applied via the lever arm of the X-Y table to center the joint. The pressure sensor was passed through the opened anterior capsule and placed between the humeral head and glenoid.

**Figure 2:**
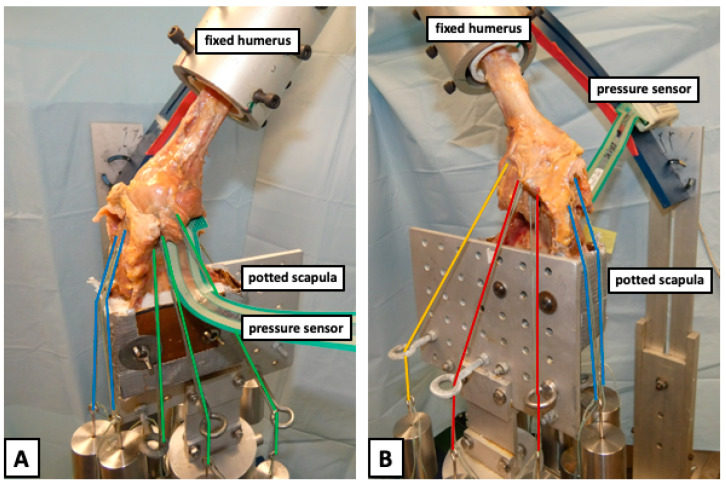
Displaying a right shoulder specimen mounted to the shoulder testing rig in 60° of abduction from the (**A**) anterior and (**B**) posterior view. The scapula was fixed to a vertical linear bearing translator and lever arm system on top of an X-Y table, allowing for glenohumeral translation in the anteroposterior and superoinferior directions. The pressure sensor was passed through the opened anterior capsule and placed between the humeral head and glenoid. The rotator cuff muscles were loaded based on physiological cross-sectional area ratios with multiple lines of pull. Specifically, two lines of pull were used for the supraspinatus (blue), three for the subscapularis (green), two for the infraspinatus (red), and one for the teres minor (yellow). Each line of pull was loaded with 5 N, resulting in a total load of 40 N.

The specimens were mounted to a validated shoulder testing rig as previously described, which allowed for positioning of the glenohumeral joint in 6 degrees of freedom (Figure 1) [21,22,23,24,25,26]. With the glenoid surface being in a horizontal position parallel to the floor, the scapula was fixed to a vertical linear bearing translator and lever arm system on top of an X-Y table, allowing for glenohumeral translation in the anteroposterior and superoinferior directions. The rotation of the humerus was defined as neutral with the bicipital groove being aligned with the anterior margin of the acromion according to Selecky et al. [20,27]. The rotator cuff muscles were loaded based on physiological cross-sectional area ratios with multiple lines of pull as previously described [28,29]. Specifically, two lines of pull were used for the supraspinatus, three for the subscapularis, two for the infraspinatus, and one for the teres minor (Figure 2) [21,29]. Each line of pull was loaded with 5 N, resulting in a total load of 40 N [21,29].

### 2.3. Surgical Technique

Total shoulder arthroplasty was performed using an anatomic stemless implant (Eclipse system, Arthrex Inc., Naples, FL, USA) according to a previously described technique [3,30]. Each surgery was performed by the same surgeon (L.N.M.), to minimize performance bias. Oriented along the specimen’s anatomic retro-torsion, two 1.6 mm K-wires were pre-drilled in line with the desired resection plane, exiting the opposite cortex at the boundary of the articular cartilage. Guided by the two K-wires, an osteotomy was performed using an oscillating saw. After measuring the anterior–posterior dimension of the resected humeral head, the size of the baseplate (trunnion) was determined. The trunnion was then fixed to the resected humeral neck and a hollow screw was inserted. The custom-made trunnion used for this study was additionally secured with a small, protruding spike, to allow the different prosthetic heads to be easily switched during testing.

Glenoid replacement was performed using a keeled glenoid system (Univers II, Arthrex Inc., Naples, FL, USA). A glenoid guide was placed on the central axis of the exposed articular surface of the glenoid, with the guide handle being oriented in line with the anatomic slope of the anterior neck. Following preparation, a keeled glenoid implant was inserted in the created slot and impacted.

### 2.4. Humeral Head Prosthetic Design

Both elliptical and spherical prosthetic humeral heads were custom-made (Arthrex Inc., Naples, FL, USA). The designs, including equations for dimension width, radius of curvature, and height, were chosen according to previously published studies [8,9]. A small hole in the undersurface allowed the humeral head implant to be securely placed on the protruding spike of the trunnion, avoiding rotation of the implant during testing and allowing heads to be easily switched between testing conditions. 

### 2.5. Biomechanical Testing

During testing, an axial compression load of 40 N was constantly applied via the lever arm of the X-Y table to center the joint [14]. Each specimen underwent the three following conditions: (1) native, and aTSA with (2) a matched-fit elliptical head and (3) a matched-fit spherical head. According to Jun et al. [14], 50° of internal and 50° of external axial rotation were alternatingly applied to the humerus at 0°, 15°, 30°, 45°, and 60° of glenohumeral abduction in the scapular plane. The testing order of glenohumeral abduction positions (0°, 15°, 30°, 45°, 60°) and head designs (elliptical or spherical) was randomly assigned to avoid bias.

Contact pressure and contact area were measured using a pressure-sensitive foil (saturation pressure, 300 PSI; pressure mapping sensor model 4205, sensel density of 27.6 sensels/cm^2^; Tekscan Inc., Boston, MA, USA) [31,32]. The sensor was passed through the opened anteroinferior capsule while preserving the anterior–inferior glenohumeral ligament, which was required for accurately visualizing the resection plane of the humeral head during surgical replacement. A separate sensor was used for each specimen. Each sensor was calibrated before and after biomechanical testing, to account for potential loss of sensitivity during testing [31,33]. Following placement of the sensor, a template of the glenoid surface of each specimen was created using the pressure mapping software (I-Scan pressure mapping system, Tekscan Inc., Boston, MA, USA) to only capture pressure changes appearing on the glenoid surface, while precluding measurement of interfering artifacts caused by the capsule or rotator cuff muscles [32].

Internal and external rotation were alternatingly applied five times for every condition. Values of each specimen were then averaged and presented as the final values. Throughout entire testing, specimens were not removed from the testing rig, nor was the testing rig disassembled. The protruding spike of the trunnion allowed us to easily switch between the elliptical and spherical head designs during testing.

### 2.6. Statistical Analysis

A power analysis was carried out to determine detectable differences in pressure, using standard deviations estimated from the literature as well as pilot data from our laboratory [34]. Assuming a common standard deviation of 15 kPa, a sample size of 6 specimens would provide 80% power to detect a 25 kPa difference in pressure at an α level of 0.05.

Descriptive statistics including mean and standard deviation (SD) as well as median and interquartile range (IQR) were calculated to characterize the groups. Differences in contact pressure and area between the implants were assessed using multilevel mixed-effects generalized linear models. A random intercept was used to account for specimens in different conditions. For each analysis, the distribution of the residual was examined and found to conform to a normal distribution. Comparisons of marginal mean values at each angle of abduction were carried out with adjustment for multiple comparisons using the Holm–Bonferroni Sequential Correction method, in case of initial statistical significance. An alpha level of 0.05 was set for all comparisons. All statistical analyses were performed using Stata 15.1 (StataCorp. 2017. Stata Statistical Software: Release 15. College Station, TX, USA: StataCorp LP).

## 3. Results

### 3.1. Contact Pressure

The elliptical head design demonstrated a significantly lower contact pressure in external rotation compared to the spherical design at 30° of abduction (Δ−111.4 kPa; 95% CI: −0.1–−221.9; *p* = 0.048; Table 1). However, there were no significant differences in contact pressure during external rotation at 0°, 15°, 45°, and 60° of abduction (*p* > 0.05, respectively). Further, elliptical and spherical head implants showed similar contact pressure in neutral rotation and internal rotation at all tested angles of abduction (*p* > 0.05, respectively). 

### 3.2. Peak Contact Pressure

The elliptical head implants showed a significantly lower peak contact pressure in external rotation compared to the spherical implants at 0° (Δ−712.0 kPa; 95% CI: −54.5–−1369.5; *p* = 0.034), 15° (Δ−894.9 kPa; 95% CI: −286.2–−1503.6; *p* = 0.004), 30° (Δ−897.7 kPa; 95% CI: −289.0–−1506.4; *p* = 0.004), and 45° (Δ−796.9 kPa; 95% CI: −188.2–−1405.6; *p* = 0.010) of abduction, while no significant difference was observed at 60° of abduction (*p* = 0.451; Table 2). Further, the elliptical and spherical head implants showed similar peak contact pressure in neutral rotation and internal rotation at all tested angles of abduction (*p* > 0.05, respectively).

### 3.3. Contact Area

The elliptical and spherical head designs demonstrated similar glenohumeral contact areas in neutral rotation, external rotation, and internal rotation at all tested angles of abduction (*p* > 0.05, respectively; Table 3).

## 4. Discussion

The most important finding of the present study was that the spherical heads showed significantly higher peak contact pressure during external rotation at 0° to 45° of glenohumeral abduction, when compared to the elliptical head implants in the setting of stemless aTSA. However, in neutral rotation and internal rotation, peak contact pressure was similar between the implant designs. Further, the finding that the spherical head implants exhibited a significantly higher contact pressure was limited to external rotation at 30° of abduction, while there were no significant differences in contact pressure between the two designs in the other motions tested. In addition, the spherical and elliptical heads showed similar contact areas during external and internal rotation as well as in neutral rotation at all abduction angles. These biomechanical observations provide further evidence regarding the time-zero effect of different humeral head implant shapes on glenohumeral contact mechanics in shoulder arthroplasty and underscore the favorable biomechanical properties of elliptical-shaped humeral head implants. Of clinical relevance, these findings may suggest that elliptical head implants hold a lower potential for abrasion along with superior durability of the components. However, comparative analyses pertaining to long-term functional and radiographic outcomes as well as complication and revision rates after spherical- or elliptical-shaped aTSA are yet to be reported.

Recently, anatomic studies have reported that the humeral head shape is more elliptical rather than a perfect sphere [11,35]. With the head assuming an elliptical shape in the anterior–posterior dimension at the periphery of the articular margin, this results in an 8–12% difference in head radius when comparing frontal and sagittal planes [11,35]. Further, previous cadaveric studies have suggested that glenohumeral mechanics can be significantly altered with a change of 4–5 mm in the articulating surface during TSA [14,36]. As this magnitude of mismatch has been shown to result from using a spherical humeral head component, this has raised concerns for the use of a spherical humeral head design as the gold standard [8,9]. Humphrey et al. recently compared spherical to elliptical head implants in a three-dimensional computational model and found that the spherical design was only capable of matching the native head (within a range of 3 mm) in 41–78% of cases, compared to 96–100% for elliptical designs, regardless of head size [9].

As, based on these previous findings, implants more closely restoring the native shoulder anatomy may ensure more physiological joint kinematics and superior durability, these findings have questioned if using a spherical implant design is most suitable to replicate the native humeral head [13]. These anatomic findings have been supported by biomechanical studies, which have shown that an elliptical implant design more accurately restored native glenohumeral joint properties in terms of kinematics, translation, and rotational range of motion [13,14]. A biomechanical study by Jun et al. compared the rotational range of motion of elliptical and spherical head implants to the native humeral head in shoulders that underwent hemiarthroplasty [13]. The authors observed no significant difference in rotational range of motion between the elliptical and native head, while the use of a spherical head design resulted in a significantly lower range of motion, especially in internal rotation in the scapular plane [13]. More specifically, a difference between the two implant designs was only observed in 30° and 60° of abduction, with the elliptical design allowed for significantly more internal rotation motion [13]. Furthermore, elliptical-shaped head implants were found to have increased glenohumeral translation during axial rotation when compared to spherical imzplants, more closely resembling native glenohumeral kinematics [14]. Interestingly, a recent biomechanical evaluation showed that increased glenohumeral translation of the humeral head implant was associated with a greater micromotion of the glenoid component during axial rotation [37].

Despite these previous investigations, the true effect of elliptical and spherical head designs on contact mechanics during dynamic range of motion in aTSA remains unknown, which may be a critical aspect for predicting implant longevity. The present study found that spherical humeral head implants resulted in significantly higher contact pressure between the articulating components during external rotation in the range of 0° to 45° of glenohumeral abduction. Interestingly, the increased peak pressure of spherical heads during external rotation was not observed at 60° of abduction. This finding may be attributable to the fact that the shoulder joint is more constrained in this position, consequently minimizing the contribution of implant shape to contact pressure. Although this study generally observed a trend of increased contact pressure of the spherical heads during external rotation, the difference compared to the elliptical heads only reached statistical significance at 30° of abduction, which may be explained by the differing conformity of the two components at various abduction positions. However, it has to be acknowledged that the pressure sensor used in this study may not be fully capable of capturing the complex shear forces occurring due to translation of the humeral head implant during axial rotation, which may be an additional critical predictor for the development of glenoid wear over time. Further changes in contact pressure may also be a result of the varying tightness of soft tissue restraints after aTSA. 

These biomechanical observations underscore the favorable biomechanical properties of elliptical-shaped humeral head implants, which have also recently been reflected in clinical studies [17,18]. Cavinatto et al. performed a radiographic evaluation of 117 patients who underwent aTSA and found that the stemless elliptical design most closely reproduced the geometry of the native humeral head when compared to the stemmed and stemless spherical design, in terms of humeral head height, prosthesis overhang, and compound reconstruction score [18]. More importantly, Budge et al. reported that stemless aTSA using a novel multiplanar osteotomy and elliptical humeral head implant resulted in significantly greater forward flexion and external rotation 6 and 12 weeks postoperatively, along with a better restoration of the radiographic center of rotation when compared with standard aTSA [17]. However, comparative studies investigating long-term functional and radiographic outcomes as well as failure rates of elliptical and spherical humeral head implants are yet to be reported.

There were several limitations to the study. Humeral head implant design may show a different effect in vivo when compared to observations during laboratory cadaveric testing. Secondly, differences in the native anatomy of each individual specimen, with the humeral head either being more elliptical or spherical in shape, may have further influenced the results. In addition, specimens with moderate to severe osteoarthritis were excluded from the study, as varying degrees of osteoarthritic changes may have limited the comparability of contact pressure measurements. Further, differing speed during internal and external rotation may have in part affected the measurements. However, care was taken to perform the rotational movement to 50° internal or external rotation in a steady and balanced way, to keep inter-cadaveric variations as low as possible. Moreover, the shoulder model of the present study was not able to account for differing tightness of the glenohumeral joint capsule, which may have inherently influenced pressure measurements. Lastly, the anteroinferior capsule was opened during surgical replacement while preserving the anterior IGHL, to accurately visualize the resection plane of the humeral head. While a capsular repair was deemed infeasible due to the necessity of frequently switching the prosthetic humeral head components during testing and placing the pressure sensor into the joint, this may have potentially affected the results.

## 5. Conclusions

In the setting of aTSA, elliptical heads showed significantly lower peak contact pressure during external rotation at 0° to 45° of abduction, when compared to spherical head implants. However, in neutral rotation and internal rotation peak contact pressure was similar between implant designs. Further, elliptical head implants were found to have a significantly lower contact pressure only in external rotation at 30° of abduction, while the remaining comparisons revealed no significant differences between the two designs. In addition, spherical and elliptical heads showed similar contact areas during external and internal rotation as well as in neutral rotation at all abduction angles.

## Figures and Tables

**Table 1:** Contact pressure (kPa) during neutral rotation, external rotation, and internal rotation. Abbreviations: IQR = interquartile range; SD = standard deviation.

			Neutral Rotation	External Rotation	Internal Rotation
**0°**	**Native**	*mean ± SD*	132.0 ± 18.2	271.3 ± 321.7	133.1 ± 66.4
		*median*	128.0	150.0	115.0
		*IQR*	6.0	177.0	80.0
	**Elliptical**	*mean ± SD*	187.8 ± 57.1	227.2 ± 160.8	211.2 ± 67.5
		*median*	192.0	182.5	189.5
		*IQR*	93.0	75.0	26.0
	**Sphere**	*mean ± SD*	220.3 ± 128.3	339.2 ± 228.0	218.3 ± 78.2
		*median*	193.5	297.5	205.5
		*IQR*	192.0	192.0	61.0
**15°**	**Native**	*mean ± SD*	123.7 ± 33.0	236.0 ± 182.0	118.0 ± 66.3
		*median*	129.5	172.5	101.5
		*IQR*	22.0	299.0	44.0
	**Elliptical**	*mean ± SD*	174.3 ± 166.8	194.0 ± 114.1	206.0 ± 56.0
		*median*	183.0	159.0	193.0
		*IQR*	51.0	63.0	88.0
	**Sphere**	*mean ± SD*	178.4 ± 56.8	289.7 ± 185.7	153.3 ± 51.9
		*median*	184.0	196.0	156.0
		*IQR*	49.0	390.0	119.0
**30°**	**Native**	*mean ± SD*	179.6 ± 115.5	225.3 ± 114.3	162.4 ± 94.3
		*median*	136.0	202.0	140.0
		*IQR*	178.0	240.0	179.0
	**Elliptical**	*mean ± SD*	259.4 ± 217.5	148.4 ± 44.6	209.9 ± 72.6
		*median*	176.0	159.0	242.0
		*IQR*	20.0	71.0	148.0
	**Sphere**	*mean ± SD*	253.3 ± 215.0	259.9 ± 207.5	262.9 ± 150.4
		*median*	177.0	279.0	199.0
		*IQR*	127.0	267.0	170.0
**45°**	**Native**	*mean ± SD*	145.1 ± 46.3	189.0 ± 115.4	124.0 ± 37.6
		*median*	126.0	156.0	123.0
		*IQR*	101.0	185.0	54.0
	**Elliptical**	*mean ± SD*	292.3 ± 139.5	191.9 ± 94.5	240.9 ± 128.1
		*median*	250.0	204.0	241.0
		*IQR*	76.0	159.0	266.0
	**Sphere**	*mean ± SD*	242.1 ± 158.3	297.7 ± 281.8	218.3 ± 157.5
		*median*	186.0	161.0	142.0
		*IQR*	280.0	304.0	136.0
**60°**	**Native**	*mean ± SD*	112.3 ± 15.8	177.6 ± 88.9	149.0 ± 50.6
		*median*	113.0	176.0	131.0
		*IQR*	18.0	71.0	104.0
	**Elliptical**	*mean ± SD*	224.0 ± 124.5	254.9 ± 140.7	174.9 ± 81.8
		*median*	224.0	266.0	155.0
		*IQR*	161.0	253.0	160.0
	**Sphere**	*mean ± SD*	253.9 ± 231.8	244.3 ± 124.5	262.4 ± 177.4
		*median*	155.0	285.0	199.0
		*IQR*	242.0	206.0	275.0

**Table 2:** Peak contact pressure (kPa) during neutral rotation, external rotation, and internal rotation. Abbreviations: IQR = interquartile range; SD = standard deviation.

			Neutral Rotation	External Rotation	Internal Rotation
**0°**	**Native**	*mean ± SD*	383.2 ± 60.5	1043.0 ± 1258.9	520.1 ± 235.0
		*median*	360.0	537.0	559.0
		*IQR*	108.0	962.0	478.0
	**Elliptical**	*mean ± SD*	1136.5 ± 1146.7	682.3 ± 343.7	1177.8 ± 663.0
		*median*	831.5	606.0	1003.5
		*IQR*	729.0	214.0	974.0
	**Sphere**	*mean ± SD*	1335.3 ± 1360.9	1394.3 ± 728.1	983.2 ± 554.8
		*median*	837.0	1225.5	778.5
		*IQR*	1805.0	776.0	737.0
**15°**	**Native**	*mean ± SD*	386.3 ± 139.0	937.7 ± 1148.9	416.7 ± 200.9
		*median*	367.0	515.0	372.5
		*IQR*	68.0	891.0	197.0
	**Elliptical**	*mean ± SD*	1179.1 ± 480.4	586.9 ± 181.8	1207.7 ± 690.4
		*median*	1108.0	559.0	1123.0
		*IQR*	947.0	329.0	1162.0
	**Sphere**	*mean ± SD*	1126.9 ± 739.3	1481.7 ± 1253.2	857.0 ± 626.5
		*median*	1149.0	1050.0	748.0
		*IQR*	892.0	2429.0	1053.0
**30°**	**Native**	*mean ± SD*	602.6 ± 385.7	794.9 ± 657.3	581.4 ± 415.1
		*median*	515.0	577.0	411.0
		*IQR*	683.0	995.0	538.0
	**Elliptical**	*mean ± SD*	1170.7 ± 282.5	593.7 ± 308.1	1140.4 ± 716.4
		*median*	1066.0	598.0	892.0
		*IQR*	311.0	471.0	1349.0
	**Sphere**	*mean ± SD*	1175.9 ± 676.3	1491.4 ± 1159.1	1275.4 ± 854.7
		*median*	995.0	1493.0	974.0
		*IQR*	1388.0	2139.0	657.0
**45°**	**Native**	*mean ± SD*	481.4 ± 177.2	682.0 ± 556.3	425.3 ± 143.8
		*median*	400.0	499.0	427.0
		*IQR*	364.0	1016.0	273.0
	**Elliptical**	*mean ± SD*	1318.9 ± 428.6	803.3 ± 733.9	1072.7 ± 886.1
		*median*	1224.0	739.0	984.0
		*IQR*	604.0	578.0	1350.0
	**Sphere**	*mean ± SD*	1366.4 ± 894.5	1600.1 ± 1297.7	1153.1 ± 906.1
		*median*	1078.0	1042.0	644.0
		*IQR*	1047.0	2135.0	1800.0
**60°**	**Native**	*mean ± SD*	356.3 ± 115.4	582.3 ± 299.7	572.7 ± 188.9
		*median*	308.0	439.0	640.0
		*IQR*	214.0	571.0	317.0
	**Elliptical**	*mean ± SD*	1119.1 ± 896.5	1322.0 ± 1071.3	801.1 ± 549.2
		*median*	688.0	888.0	743.0
		*IQR*	1136.0	2202.0	1259.0
	**Sphere**	*mean ± SD*	1104.6 ± 1049.2	1088.1 ± 745.9	1213.4 ± 784.1
		*median*	733.0	1082.0	1375.0
		*IQR*	2027.0	1129.0	1519.0

**Table 3:** Contact area (mm^2^) during neutral rotation, external rotation, and internal rotation. Abbreviations: IQR = interquartile range; SD = standard deviation.

			Neutral Rotation	External Rotation	Internal Rotation
**0°**	**Native**	*mean ± SD*	328.6 ± 119.8	203.0 ± 67.3	270.7 ± 97.5
		*median*	312.0	203.0	312.0
		*IQR*	207.0	73.0	181.0
	**Elliptical**	*mean ± SD*	203.2 ± 52.9	110.7 ± 63.6	158.0 ± 83.7
		*median*	198.0	88.5	143.5
		*IQR*	62.0	55.0	123.0
	**Sphere**	*mean ± SD*	177.2 ± 50.2	102.2 ± 40.9	143.2 ± 79.0
		*median*	183.5	94.0	128.5
		*IQR*	73.0	66.0	123.0
**15°**	**Native**	*mean ± SD*	312.0 ± 78.3	196.5 ± 97.1	242.8 ± 76.3
		*median*	301.0	197.5	249.5
		*IQR*	95.0	116.0	83.0
	**Elliptical**	*mean ± SD*	155.0 ± 56.7	93.6 ± 46.1	117.1 ± 64.5
		*median*	149.0	87.0	98.0
		*IQR*	65.0	69.0	118.0
	**Sphere**	*mean ± SD*	202.7 ± 56.4	107.3 ± 53.4	122.3 ± 39.8
		*median*	200.0	116.0	98.0
		*IQR*	62.0	109.0	83.0
**30°**	**Native**	*mean ± SD*	257.3 ± 98.2	182.9 ± 114.4	226.6 ± 77.3
		*median*	258.0	196.0	261.0
		*IQR*	84.0	221.0	156.0
	**Elliptical**	*mean ± SD*	153.9 ± 64.3	114.9 ± 65.7	117.9 ± 39.2
		*median*	178.0	92.0	124.0
		*IQR*	101.0	134.0	47.0
	**Sphere**	*mean ± SD*	166.0 ± 65.7	105.7 ± 63.3	115.4 ± 57.1
		*median*	178.0	94.0	116.0
		*IQR*	83.0	109.0	84.0
**45°**	**Native**	*mean ± SD*	361.9 ± 164.4	260.3 ± 140.4	293.4 ± 106.9
		*median*	305.0	265.0	261.0
		*IQR*	316.0	291.0	123.0
	**Elliptical**	*mean ± SD*	131.4 ± 61.2	96.9 ± 51.8	109.0 ± 52.5
		*median*	120.0	80.0	112.0
		*IQR*	62.0	76.0	43.0
	**Sphere**	*mean ± SD*	146.6 ± 49.5	105.3 ± 70.4	125.1 ± 64.8
		*median*	149.0	105.0	112.0
		*IQR*	29.0	69.0	105.0
**60°**	**Native**	*mean ± SD*	397.4 ± 108.4	290.6 ± 111.7	357.1 ± 128.4
		*median*	363.0	299.0	368.0
		*IQR*	58.0	254.0	156.0
	**Elliptical**	*mean ± SD*	123.3 ± 56.7	118.0 ± 61.9	114.7 ± 61.1
		*median*	112.0	120.0	102.0
		*IQR*	44.0	77.0	36.0
	**Sphere**	*mean ± SD*	144.6 ± 80.7	98.4 ± 75.9	103.6 ± 63.5
		*median*	152.0	58.0	102.0
		*IQR*	171.0	123.0	80.0

## Data Availability

The datasets used and/or analyzed during the current study are available from the corresponding author on reasonable request.

## References

[1] Bryant D., Litchfield R., Sandow M., Gartsman G.M., Guyatt G., Kirkley A. (2005). A comparison of pain, strength, range of motion, and functional outcomes after hemiarthroplasty and total shoulder arthroplasty in patients with osteoarthritis of the shoulder. A systematic review and meta-analysis. J. Bone Jt. Surg. Am..

[2] Lo I.K., Litchfield R., Griffin S., Faber K., Patterson S.D., Kirkley A. (2005). Quality-of-life outcome following hemiarthroplasty or total shoulder arthroplasty in patients with osteoarthritis. A prospective, randomized trial. J. Bone Jt. Surg. Am..

[3] Hawi N., Magosch P., Tauber M., Lichtenberg S., Habermeyer P. (2017). Nine-year outcome after anatomic stemless shoulder prosthesis: Clinical and radiologic results. J. Shoulder Elb. Surg..

[4] Bigliani L.U., Kelkar R., Flatow E.L., Pollock R.G., Mow V.C. (1996). Glenohumeral stability. Biomechanical properties of passive and active stabilizers. Clin. Orthop. Relat. Res..

[5] Karduna A.R., Williams G.R., Williams J.L., Iannotti J.P. (1996). Kinematics of the glenohumeral joint: Influences of muscle forces, ligamentous constraints, and articular geometry. J. Orthop. Res..

[6] Kelkar R., Wang V.M., Flatow E.L., Newton P.M., Ateshian G., Bigliani L.U., Pawluk R.J., Mow V.C. (2001). Glenohumeral mechanics: A study of articular geometry, contact, and kinematics. J. Shoulder Elb. Surg..

[7] Soslowsky L.J., Flatow E.L., Bigliani L.U., Mow V.C. (1992). Articular geometry of the glenohumeral joint. Clin. Orthop. Relat. Res..

[8] Humphrey C.S., Sears B.W., Curtin M.J. (2016). An anthropometric analysis to derive formulae for calculating the dimensions of anatomically shaped humeral heads. J. Shoulder Elb. Surg..

[9] Humphrey C.S., Gale A.L. (2018). Spherical versus elliptical prosthetic humeral heads: A comparison of anatomic fit. J. Shoulder Elbow Surg..

[10] Habermeyer P., Magosch P., Weiss C., Hawi N., Lichtenberg S., Tauber M., Ipach B. (2017). Classification of humeral head pathomorphology in primary osteoarthritis: A radiographic and in vivo photographic analysis. J. Shoulder Elb. Surg..

[11] Iannotti J.P., Gabriel J.P., Schneck S.L., Evans B.G., Misra S. (1992). The normal glenohumeral relationships. An anatomical study of one hundred and forty shoulders. J. Bone Jt. Surg. Am..

[12] Harrold F., Wigderowitz C. (2013). Humeral head arthroplasty and its ability to restore original humeral head geometry. J. Shoulder Elb. Surg..

[13] Jun B.J., Iannotti J.P., McGarry M.H., Yoo J.C., Quigley R.J., Lee T.Q. (2013). The effects of prosthetic humeral head shape on glenohumeral joint kinematics: A comparison of non-spherical and spherical prosthetic heads to the native humeral head. J. Shoulder Elb. Surg..

[14] Jun B.J., Lee T.Q., McGarry M.H., Quigley R.J., Shin S.J., Iannotti J.P. (2016). The effects of prosthetic humeral head shape on glenohumeral joint kinematics during humeral axial rotation in total shoulder arthroplasty. J. Shoulder Elb. Surg..

[15] Muench L.N., Otto A., Kia C., Obopilwe E., Cote M.P., Imhoff A.B., Beitzel K., Mazzocca A.D., Mehl J. (2020). Rotational range of motion of elliptical and spherical heads in shoulder arthroplasty: A dynamic biomechanical evaluation. Arch. Orthop. Trauma Surg..

[16] Spangenberg G.W., Faber K.J., Langohr G.D.G., Reeves J.M. (2023). The sizing and suitability of nonspherical ellipsoid humeral heads for total shoulder arthroplasty. J. Shoulder Elb. Surg..

[17] Budge M.D., Orvets N. (2023). Stemless total shoulder arthroplasty using a novel multiplanar osteotomy and elliptical humeral head results in both improved early range of motion and radiographic center of rotation compared with standard total shoulder arthroplasty. J. Shoulder Elb. Surg..

[18] Cavinatto L., Khatib O., Martusiewicz A., Koueiter D.M., Wiater B.P., Wiater J.M. (2021). Radiographic evaluation of humeral head reconstruction with stemmed and stemless spherical implants compared with stemless elliptical head implants. JSES Int..

[19] Shapiro T.A., McGarry M.H., Gupta R., Lee Y.S., Lee T.Q. (2007). Biomechanical effects of glenoid retroversion in total shoulder arthroplasty. J. Shoulder Elb. Surg..

[20] Imhoff F.B., Camenzind R.S., Obopilwe E., Cote M.P., Mehl J., Beitzel K., Imhoff A.B., Mazzocca A.D., Arciero R.A., Dyrna F.G.E. (2019). Glenoid retroversion is an important factor for humeral head centration and the biomechanics of posterior shoulder stability. Knee Surg. Sports Traumatol. Arthrosc..

[21] Mehl J., Otto A., Imhoff F.B., Murphy M., Dyrna F., Obopilwe E., Cote M., Ladermann A., Collin P., Beitzel K. (2019). Dynamic Anterior Shoulder Stabilization with the Long Head of the Biceps Tendon: A Biomechanical Study. Am. J. Sports Med..

[22] Muench L.N., Murphey M., Oei B., Kia C., Obopilwe E., Cote M.P., Mazzocca A.D., Berthold D.P. (2023). Elliptical and spherical heads show similar obligate glenohumeral translation during axial rotation in total shoulder arthroplasty. BMC Musculoskelet. Disord..

[23] Arciero R.A., Parrino A., Bernhardson A.S., Diaz-Doran V., Obopilwe E., Cote M.P., Golijanin P., Mazzocca A.D., Provencher M.T. (2015). The effect of a combined glenoid and Hill-Sachs defect on glenohumeral stability: A biomechanical cadaveric study using 3-dimensional modeling of 142 patients. Am. J. Sports Med..

[24] Mihata T., Lee Y., McGarry M.H., Abe M., Lee T.Q. (2004). Excessive humeral external rotation results in increased shoulder laxity. Am. J. Sports Med..

[25] Pauzenberger L., Dyrna F., Obopilwe E., Heuberer P.R., Arciero R.A., Anderl W., Mazzocca A.D. (2017). Biomechanical Evaluation of Glenoid Reconstruction with an Implant-Free J-Bone Graft for Anterior Glenoid Bone Loss. Am. J. Sports Med..

[26] Schneider D.J., Tibone J.E., McGarry M.H., Grossman M.G., Veneziani S., Lee T.Q. (2005). Biomechanical evaluation after five and ten millimeter anterior glenohumeral capsulorrhaphy using a novel shoulder model of increased laxity. J. Shoulder Elb. Surg..

[27] Selecky M.T., Tibone J.E., Yang B.Y., McMahon P.J., Lee T.Q. (2003). Glenohumeral joint translation after arthroscopic thermal capsuloplasty of the posterior capsule. J. Shoulder Elb. Surg..

[28] Veeger H.E., Van der Helm F.C., Van der Woude L.H., Pronk G.M., Rozendal R.H. (1991). Inertia and muscle contraction parameters for musculoskeletal modelling of the shoulder mechanism. J. Biomech..

[29] Barrett Payne W., Kleiner M.T., McGarry M.H., Tibone J.E., Lee T.Q. (2016). Biomechanical comparison of the Latarjet procedure with and without a coracoid bone block. Knee Surg. Sports Traumatol. Arthrosc..

[30] Habermeyer P., Lichtenberg S., Tauber M., Magosch P. (2015). Midterm results of stemless shoulder arthroplasty: A prospective study. J. Shoulder Elb. Surg..

[31] Lin T., Javidan P., McGarry M.H., Gonzalez-Lomas G., Limpisvasti O., Lee T.Q. (2013). Glenohumeral contact pressure in a simulated active compression test using cadaveric shoulders. J. Shoulder Elb. Surg..

[32] Muench L.N., Berthold D.P., Otto A., Dyrna F., Bell R., Obopilwe E., Cote M.P., Imhoff A.B., Mazzocca A.D., Beitzel K. (2021). Increased Glenohumeral Joint Loads Due to a Supraspinatus Tear Can Be Reversed with Rotator Cuff Repair: A Biomechanical Investigation. Arthroscopy.

[33] Wilharm A., Hurschler C., Dermitas T., Bohnsack M. (2013). Use of Tekscan K-scan sensors for retropatellar pressure measurement avoiding errors during implantation and the effects of shear forces on the measurement precision. Biomed. Res. Int..

[34] Kim H.M., Chacon A.C., Andrews S.H., Roush E.P., Cho E., Conaway W.K., Kunselman A.R., Lewis G.S. (2016). Biomechanical benefits of anterior offsetting of humeral head component in posteriorly unstable total shoulder arthroplasty: A cadaveric study. J. Orthop. Res..

[35] Hertel R., Knothe U., Ballmer F.T. (2002). Geometry of the proximal humerus and implications for prosthetic design. J. Shoulder Elb. Surg..

[36] Harryman D.T., Sidles J.A., Harris S.L., Lippitt S.B., Matsen F.A. (1995). The effect of articular conformity and the size of the humeral head component on laxity and motion after glenohumeral arthroplasty. A study in cadavera. J. Bone Jt. Surg. Am..

[37] Muench L.N., Kia C., Murphey M., Obopilwe E., Cote M.P., Imhoff A.B., Mazzocca A.D., Berthold D.P. (2021). Elliptical heads result in increased glenohumeral translation along with micro-motion of the glenoid component during axial rotation in total shoulder arthroplasty. Arch. Orthop. Trauma Surg..

